# Comparative pathology of dog and human prostate cancer

**DOI:** 10.1002/vms3.642

**Published:** 2021-10-10

**Authors:** Toby Ryman‐Tubb, Jennifer H. Lothion‐Roy, Veronika M. Metzler, Anna E. Harris, Brian D. Robinson, Albert A. Rizvanov, Jennie N. Jeyapalan, Victoria H. James, Gary England, Catrin S. Rutland, Jenny L. Persson, Lukas Kenner, Mark A. Rubin, Nigel P. Mongan, Simone de Brot

**Affiliations:** ^1^ BioDiscovery Institute School of Veterinary Medicine and Science University of Nottingham Nottingham UK; ^2^ Department of Pathology Weill Cornell Medicine NY New York USA; ^3^ Institute of Fundamental Medicine and Science Kazan Federal University Kazan Tatarstan Russia; ^4^ Department of Molecular Biology Umeå Universitet Umeå Sweden; ^5^ Department of Biomedical Sciences Malmö Universitet Malmö Sweden; ^6^ Department of Experimental Pathology Laboratory Animal Pathology Medical University Wien Vienna Austria; ^7^ Bern Center for Precision Medicine University of Bern and Inselspital Bern Switzerland; ^8^ Department of BioMedical Research University of Bern and Inselspital Bern Switzerland; ^9^ Department of Pharmacology Weill Cornell Medicine New York New York USA; ^10^ COMPATH, Institute of Animal Pathology University of Bern Bern Switzerland

**Keywords:** adenocarcinoma, animal model, canine, neuroendocrine, urology

## Abstract

Though relatively rare in dogs, prostate cancer (PCa) is the most common non‐cutaneous cancer in men. Human and canine prostate glands share many functional, anatomical and physiological features. Due to these similarities, canine PCa has been proposed as a model for PCa in men. PCa is typically androgen‐dependent at diagnosis in men and for this reason, androgen deprivation therapies (ADT) are important treatments for advanced PCa in men. In contrast, there is some evidence that PCa is diagnosed more commonly in castrate dogs, at which point, limited therapeutic options are available. In men, a major limitation of current ADT is that progression to a lethal and incurable form of PCa, termed castrate‐resistant prostate cancer (CRPC), is common. There is, therefore, an urgent need for a better understanding of the mechanism of PCa initiation and progression to CRPC to enable the development of novel therapeutic approaches. This review focuses on the functional, physiological, endocrine and histopathological similarities and differences in the prostate gland of these species. In particular, we focus on common physiological roles for androgen signalling in the prostate of men and dogs, we review the short‐ and longer‐term effects of castration on PCa incidence and progression in the dog and relate how this knowledge may be relevant to understanding the mechanisms of CRPC in men.

## Graphical abstract

Only three species, men, dogs and lions, are believed to develop prostate cancer. Here, we review the common and distinct features of prostate cancer in men and dogs, with a view to better understanding the disease in both species.

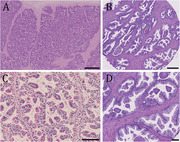

## 1 INTRODUCTION

Prostate cancer (PCa) remains a major clinical challenge. It is the most common non‐cutaneous malignancy affecting men and is estimated to have led to 31,620 deaths and 1,74,650 new cases in the USA alone in 2019 (Siegel et al., 2019). The initiation and progression of PCa in men is androgen driven (Cai et al., 2011; Sharma et al., 2013; Wang et al., 2009). For this reason, it has long been recognised that advanced PCa can be treated by ‘depriving’ PCa cells of androgen stimulation (Huggins et al., 1941). Thus, the inhibition of androgen synthesis and the pharmacological antagonism of androgen receptor (AR) function are the basis of androgen deprivation therapies (ADT) (de Bono et al., 2011; Scher et al., 2012; Sharifi et al., 2005). A major limitation of current ADT is that they often remain effective for a limited duration before patients progress to a lethal and incurable form of PCa, termed castrate‐resistant prostate cancer (CRPC) (Chandrasekar et al., 2015), where the AR continues to orchestrate pro‐oncogenic signalling (Sharma et al., 2013). The emergence of ADT‐resistance and disease progression, and the increasing numbers of treatment‐emergent neuroendocrine prostate cancers (NEPC) which lack AR expression (Dang et al., 2015; Humphrey, 2012; Mucci et al., 2000), represent major therapeutic challenges (Beltran et al., 2014; Hu et al., 2002). For these reasons, there is an urgent need to better understand the mechanisms of PCa initiation, progression to CRPC and the emergence of ADT resistance.

## 2 COMPARATIVE PROSTATE ANATOMY AND FUNCTION

The prostate plays an essential role in male fertility. Its main function is to secrete fluid which accounts for one third of the total volume of the semen. This mildly acidic fluid contains various enzymes, including PSA/KLK3 and zinc, which is crucial for semen liquefaction and motility (Huggins & Neal, 1942; Sørensen et al., 1999; Yoshida et al., 2008). The prostate gland in men is commonly described as a walnut‐sized organ lying behind the pubic symphysis at the base of the bladder with the urethra running through its centre (Mangera et al., 2013). The epithelium of the acini and ducts are composed of basal, secretory and neuroendocrine (NE) cells (Bonkhoff & Remberger, 1996; Di Sant'Agnese, 1992; McNeal, 1988). The pre‐pubertal prostate weighs approximately 2 g in boys until increasing pubertal androgen levels, stimulating prostate growth to approximately 20 g in the healthy adult male until 50 years of age, when the incidence of benign prostatic hyperplasia increases (Vickman et al., 2019). The arterial supply of the human prostate originates from the prostatic branch of the vesical artery. The venous supply is provided by the periprostatic venous plexus and communicates with the vesicoprostatic plexus which lies between the bladder and the prostate (Mangera et al., 2013). The prostate itself is heterogenous, composed of 70% glandular and 30% fibromuscular tissue encapsulated by a thick fibrous capsule (Mangera et al., 2013; McNeal, 1981). In men, the prostate gland is composed of four different zones, the central zone (CZ), transition zone (TZ), peripheral zone (PZ) and anterior zone (AZ) (McNeal, 1988). The prostate can also be divided into the base (superior/upper third of the gland, adjacent to the bladder), the mid‐prostate (middle third) containing the verumontanum, also called seminal colliculus, where the ejaculatory ducts enter the urethra and the apex (inferior/lower third) (Bhavsar & Verma, 2014). The PZ makes up approximately 70% of the prostate. Diseases, like chronic prostatitis, post‐inflammatory atrophy and cancer, most commonly arise in the PZ (De Marzo et al., 1999; McNeal, 1988). The PZ comprises many ducts, acini and some smooth muscle tissue (Bhavsar & Verma, 2014). The CZ is situated between the PZ and TZ and accounts for approximately 25% of the gland. It is cone‐shaped, surrounds the ejaculatory ducts and gets thinner at the verumontanum. The TZ makes up only approximately 5% of the gland, surrounds the urethra, and is enlarged in patients with benign prostatic hyperplasia. The AZ does not contain any glandular structures, only fibrous and smooth muscular tissue. It is the connection point to the pelvic diaphragm and partially covers the prostate as a thin fibrous capsule (Bhavsar & Verma, 2014).

**FIGURE 1:**
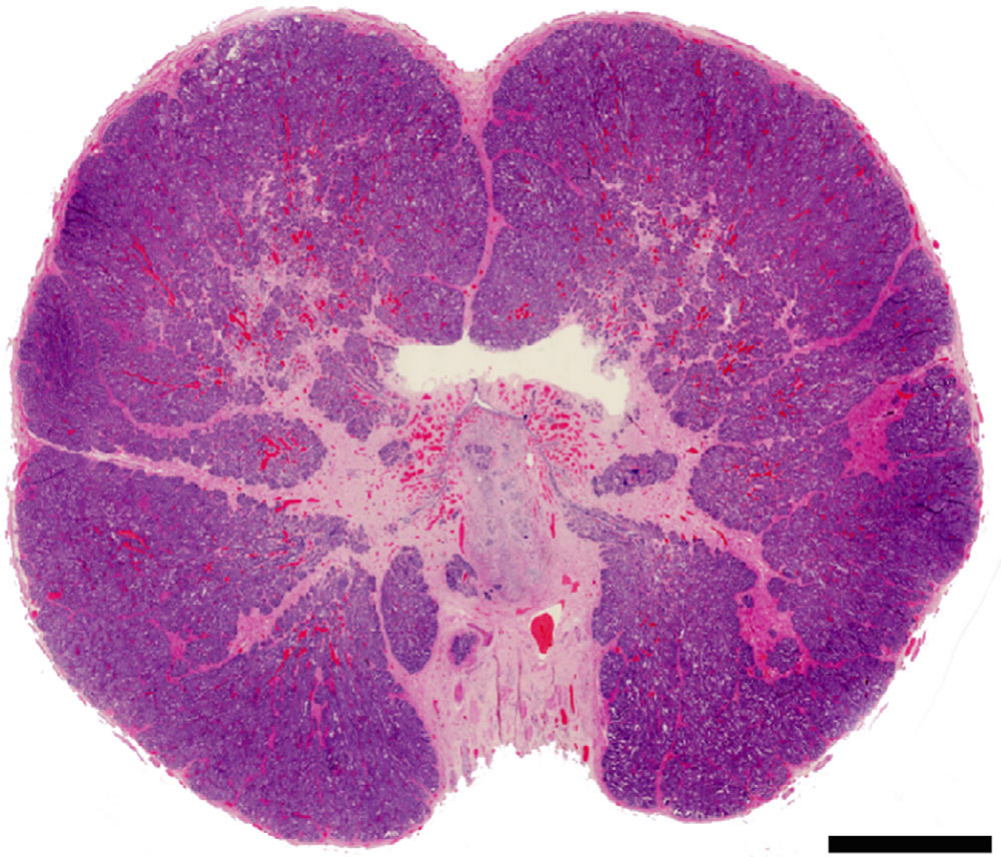
Microphotograph. Cross‐section of a benign canine prostate, which is characterized by a bilobed structure and densely packed glandular tissue. Haematoxylin and Eosin (HE) stain. Size bar indicates 4 mm

**FIGURE 2:**
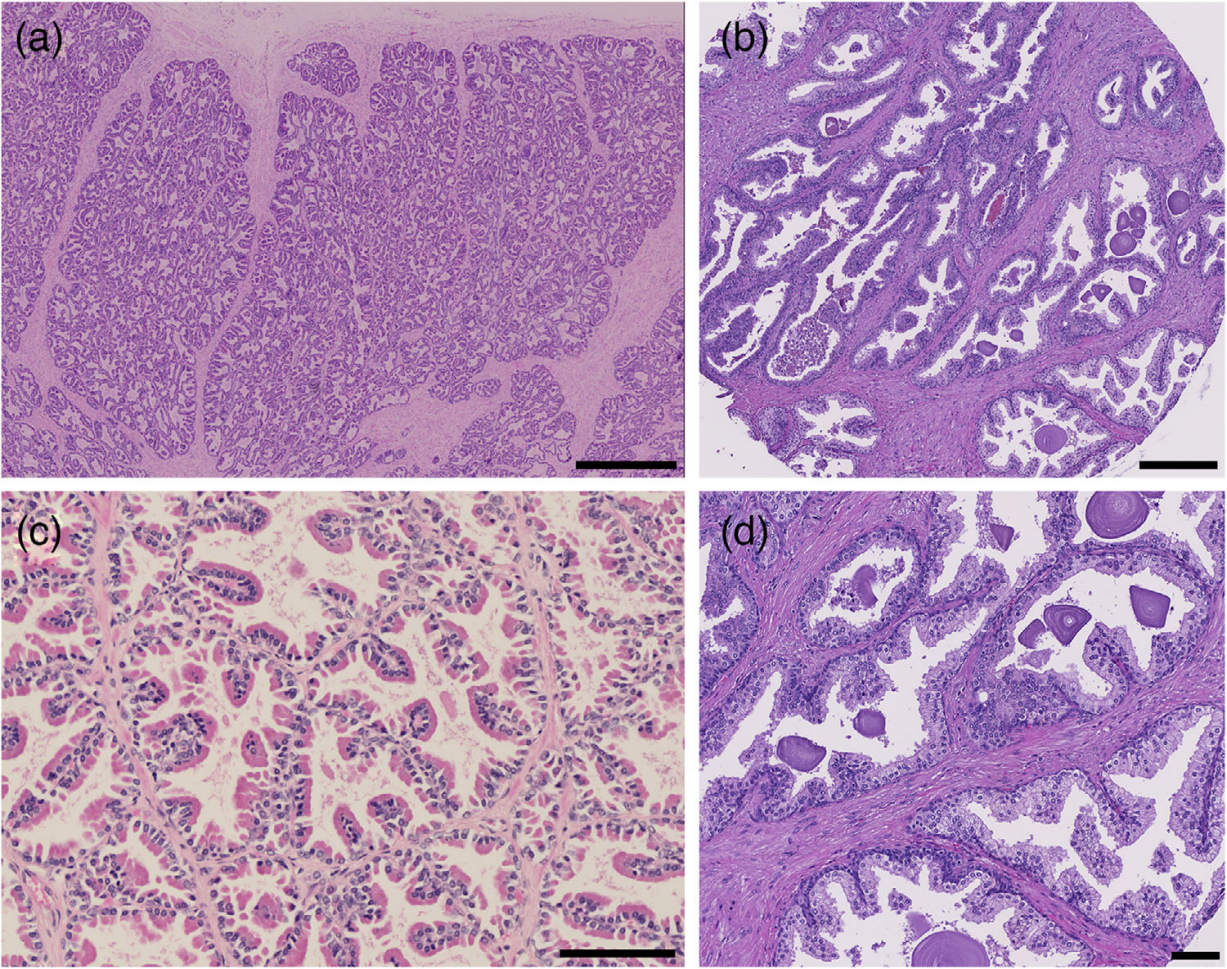
Histology of the normal adult human and canine prostate. HE stain. (a) Canine. Glandular tissue is separated by fine to moderately dense stromal projections. Size bar indicates 1 mm. (b) Human. Glands are surrounded by non‐glandular, fibromuscular stroma. Size bar indicates 300 μm. (c) Canine. Acini present with papillary projections, lined by a single layer of tall columnar epithelial cells with a deeply eosinophilic cytoplasm and basally located nuclei, supported by fine fibrovascular stroma. Basal cells are ill defined on HE‐stained tissue sections. Size bar indicates 100 μm. (d) Human. Acinar secretory cells are columnar and are separated from the basement membrane and stroma by a layer of basal cells. Intraluminal concentric lamellate bodies, referred to as corpora amylacea are a common finding in men. Size bar indicates 80 μm

As the anatomy and histology of the human prostate is familiar, in this review, we will focus primarily on the similarities and differences between the human and canine prostate. The canine prostate shares many anatomical, histological, physiological and functional similarities with that of the human prostate, and the dog is one of the few species that regularly develops spontaneous prostatic neoplasia (Huggins, 1943; LeRoy & Northrup, 2009). In most aspects, the histological appearance of the prostate gland parenchyma and stroma is very similar between men and dogs (Figures 1 and 2). This becomes even more evident when comparing the human prostate with the gland in other animal species, notably rodents (Roy‐Burman et al., 2004), which are commonly used in experimental models but have distinct prostate anatomy (Civenni et al., 2018).

As in men, the function and physiology of the canine prostate involves the secretions of various constituents of seminal plasma via its communication with the urethra (Fernando Leis‐Filho & Fonseca‐Alves, 2019). In dogs, the prostate gland is situated within the pelvic cavity, ventral (anterior) to the rectum and cranial (superior) to the pelvic symphysis (Evans et al., 1993). The canine prostate contains the urethra centrally and is surrounded by a thin fibrous tissue capsule.

**FIGURE 3:**
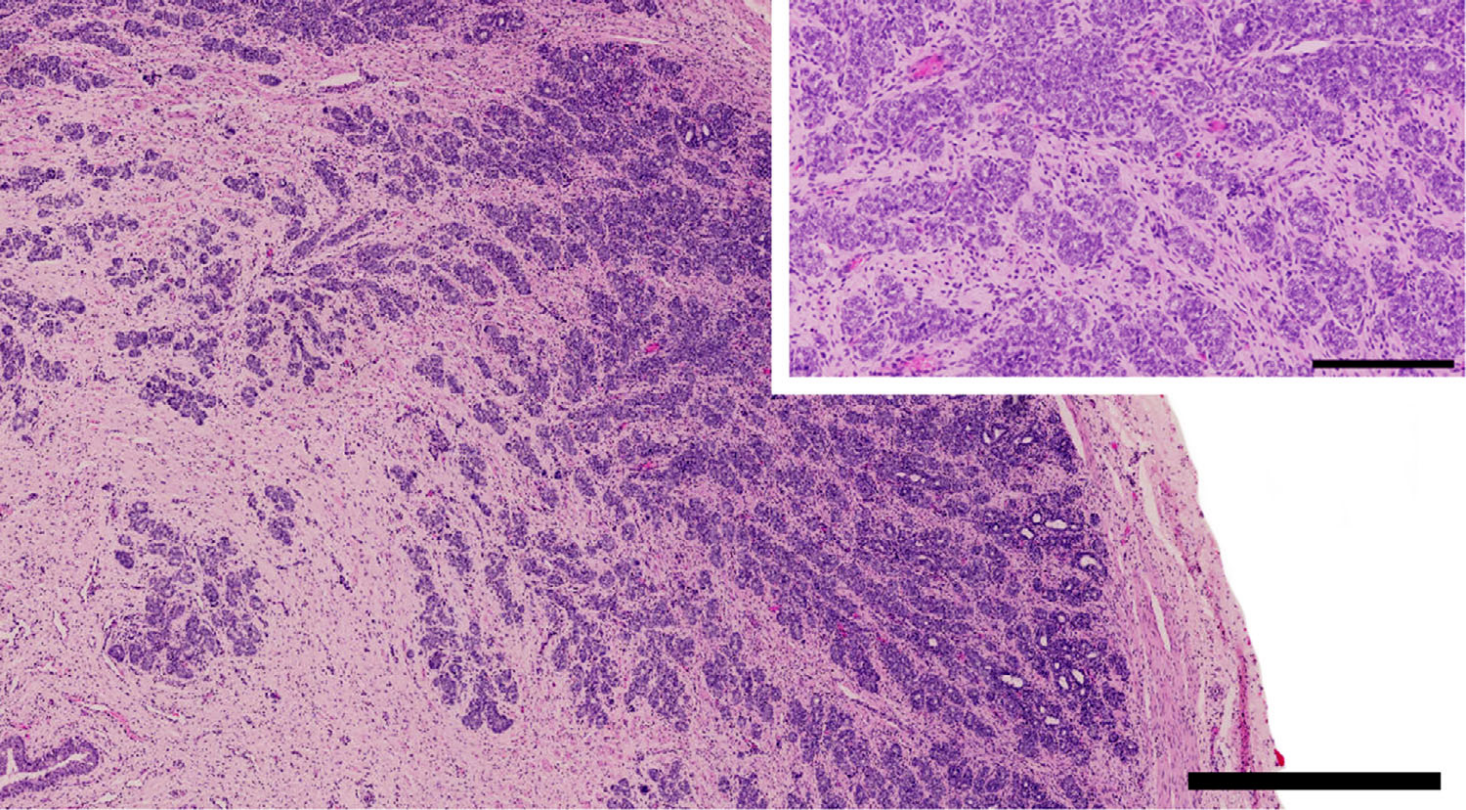
Pre‐pubertal canine prostate gland from a 3‐month‐old male entire Labrador retriever dog. Glandular tissue is characterized mainly by dense epithelial aggregates, lacking lumen formations, surrounded by dense stromal tissue. Note the multifocal small glandular lumina in the periphery of the prostate in this case. Size bar indicates 900 μm. Inset: Closer view. Size bar indicates 200 μm. HE stain

Grossly, the canine and human prostate have a similar ovoid, bilobed structure and are both situated at the base of the bladder, encompassing the proximal urethra (LeRoy & Northrup, 2009). In both species, prostatic growth and development are dependent on testicular androgen control. The size and morphology of the gland can vary with canine breed and body size, with reported mean volumes of 10–92 cm^3^ in uncastrated dogs. A distinct feature is found in neonatal puppies, where a series of long main ducts radiate from the prostatic urethra to the outer periphery of the gland. Some of these ducts contain a lumen whilst others are solid structures (Leav et al., 2001). Also, at this early developmental stage, epithelial cells that later become secretory acinar cells begin as solid aggregates surrounding small branches of the ducts. The abundant inverting stroma that separates the duct and acinar structures into lobules is highly cellular (Leav et al., 2001). The pre‐pubertal canine prostate is a lobular gland and consists mainly of dense epithelial aggregates, with a lack of lumen formations, surrounded by a thick proliferation of stromal tissue (LeRoy & Northrup, 2009) (Figure 3). Post‐puberty, the fibrous connective tissue is replaced by prostatic epithelium under androgen control (LeRoy & Northrup, 2009). Paired ductus deferens enters both lobes of the prostate on the craniolateral (superolateral) aspect running in a caudoventral (anteroinferior) direction before entering the urethra adjacent to the colliculus seminalis. The prostatic ducts within each lobe course toward the urethra and run throughout its circumference (Kutzler & Yeager, 2005). The lobes are divided by the medial septum and each lobe itself is divided further into lobules by trabeculae, with tubuloalveolar glands secreting into the urethra via small ducts (Evans et al., 1993). Arterial supply to the prostate is via the prostatic artery entering the prostatic capsule on the dorsal (posterior) surface. The venous supply to the prostate is via the prostatic and urethral veins, with lymphatic drainage to the iliac lymph nodes (Evans et al., 1993).

One major difference is that the canine prostate is histologically and morphologically homogenous, in contrast to the human prostate which harbours four distinct anatomical zones as noted earlier. In the canine prostate, 15 lobules containing glandular secretory tissue are separated by stromal projections known as capsular trabeculae (Fernando Leis‐Filho & Fonseca‐Alves, 2019). The majority of the prostate is made up of glandular secretory tissue lined with columnar epithelium (Figures 1 and 2) (Fernando Leis‐Filho & Fonseca‐Alves, 2019). Similar to the gland in men, the normal canine prostate epithelium is composed of three cell types: basal, secretory and NE (Ismail et al., 2002). The basal cell layer, however, differs between the two species with a discontinuous layer seen in dogs, and a continuous basal cell lining in the glandular acini of men. Indeed, a discontinuous basal cell layer is a strong indicator of PCa in men (Hameed & Humphrey, 2005). Secretory cuboidal to columnar epithelial cells make up the majority of the epithelial cells and line tubuloalveolar glands distributed within the lobules that drain into the small ducts surrounding the urethra (Sun et al., 2017). The third type of acinar cell, NE cells, have been reported to closely resemble those found in humans in both morphology and distribution (Ismail et al., 2002). NE cells are intraepithelial cells distributed throughout the prostate which make up a small proportion of the total epithelial population (Di Sant'Agnese, 1992). These rare cells are believed to regulate the exocrine secretion process in addition to controlling differentiation and growth of the prostate (Di Sant'Agnese, 1992; Ismail et al., 2002). NE cells in both species are regulated independently of basal and secretory cell differentiation (Ismail et al., 2002). In the castrated dog, the removal of androgens appears to cause a significant increase in NE cell density (Ismail et al., 2002). Smooth muscle is a normal stromal component of the canine prostate but to a lesser extent than what is seen in the human prostate (Sun et al., 2017).

## 3 FUNCTION AND PHYSIOLOGICAL REGULATION OF THE PROSTATE IN MEN AND DOGS

Unlike in men, the exact function(s) of the canine prostate is not fully understood. It is known that, as in men, a primary function of the canine prostate is to produce prostatic fluid which aids sperm viability and transport during ejaculation (Barsanti & Finco, 1986). Canine ejaculate consists of three fractions and prostatic fluid is present in the first and last of these fractions (England et al., 1990; Nöthling et al., 2005). This prostatic fluid is responsible for over 90% of the ejaculate volume in the dog (Basinger et al., 2003). These secretions have been shown to have a limited impact on fertility in the canine species and, unlike in humans and rodents, the prostatic fluid is not produced in seminal vesicles as these are absent in the dog (Hayward & Cunha, 2000). Another difference in dogs compared to other species is that the prostatic fluid lacks simple sugars, with the energy source for sperm remaining elusive (Smith, 2008). Canine prostatic fluid is known to contain cholesterol, citrate and lactate (Evans et al., 1993). Of the proteins within the fluid, canine prostate‐specific arginine esterase is the most abundant, comprising up to 90% of seminal proteins and is, hence, used as a marker of prostatic secretions (Gobello et al., 2002).

**FIGURE 4:**
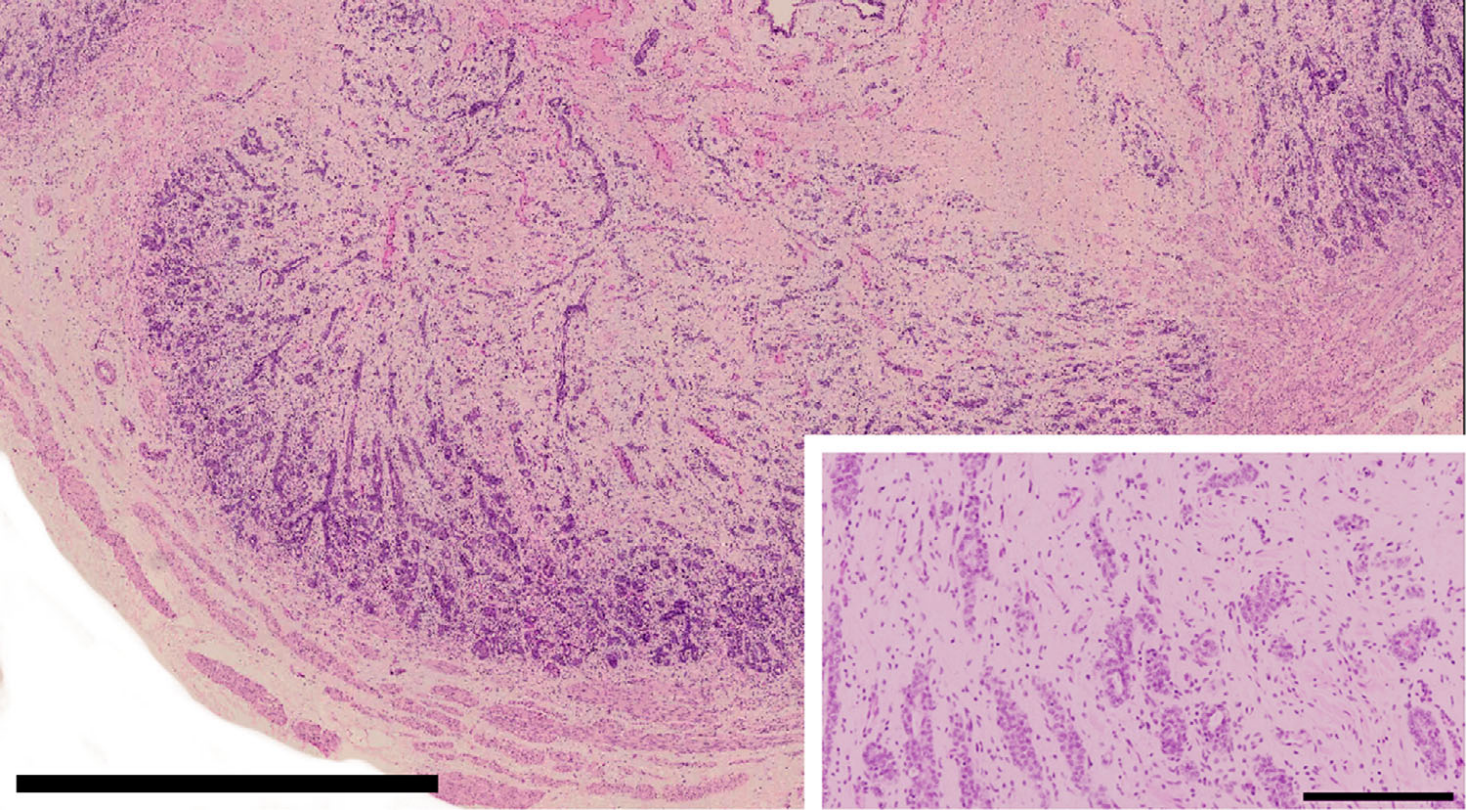
Canine prostate with diffuse glandular atrophy due to surgical castration. Size bar indicates 2 mm. Inset: Closer view. Size bar indicates 200 μm. HE stain

The canine prostate, as in other species, is under androgen control, acting via the AR. Androgens play a key role in the development, growth and maintenance of the prostate, affecting proliferation and differentiation of luminal epithelial cells (Sun et al., 2017). It is known that the level of androgen hormones is directly correlated to the secretory function and volume of the prostate (Fernando Leis‐Filho & Fonseca‐Alves, 2019). Following castration of dogs, the androgen supply is reduced to less than 1 ng/mL and significant atrophy of the prostate is seen, primarily within the acinar structures (Lai et al., 2008; Vanderstichel et al., 2015) (Figure 4). As atrophy advances, only a single epithelial layer of ductular and acinar structures remains within the prostate. In these cases, it is nearly impossible to distinguish between ducts and atrophic acini via light microscopy (Lai et al., 2008). However, even in glands with diffuse atrophy, the lobular structure remains and is recognised by the interstitial stroma. Castration also appears to affect the expression of certain markers released within the prostate and causes an increase in the number of cells with a ductal phenotype, the significance of which is unknown (Lai et al., 2008).

## 4 PROSTATIC DISEASE IN MEN AND DOGS

As noted earlier, PCa continues to be a major clinical challenge for men, and metastatic PCa remains incurable. For this reason, there is an urgent need to better understand the molecular mechanisms underpinning PCa initiation and progression. While sophisticated transgenic mouse models of PCa have advanced understanding of the disease, as reviewed by Civenni et al. (2018), there is a considerable interest in canine PCa as a model of spontaneous disease (LeRoy & Northrup, 2009; Sun et al., 2017). Indeed, the biology of prostate disease shares some striking similarities between these species. For example, canine prostate neoplasia is influenced by a variety of factors, notably the removal of androgens via castration, although the exact influence of castration on progression and incidence of the disease remains controversial (Schrank & Romagnoli, 2020). Given the increasing importance of ADT on emergent NEPC in men (Aggarwal et al., 2018), there is increasing interest in the pathological mechanisms of castration‐related PCa in dogs, with a view to better understanding the disease in men.

A study by Krawiec and Heflin (1992) reported that prostate disease accounts for approximately 3% of male‐entire dog veterinary cases. The most prevalent diseases of the canine prostate include benign prostatic hyperplasia (BPH), prostatitis, prostatic neoplasia and prostatic cysts. BPH is by far the most common disease process seen in the canine prostate accounting for over 50% of cases, with prostatitis (infectious or non‐infectious) responsible for 20%, and neoplasia accounting for only approximately 7% of cases seen (Krawiec & Heflin, 1992; Lévy et al., 2014). Canine prostatic neoplasia diagnoses are much less common in dogs (0.35%) as compared within men (∼8‐25% depending on ethnicity) (Lloyd et al., 2015; Schrank & Romagnoli, 2020; Weaver, 1981). This difference in incidence may at least be partially attributable to the likely underdiagnosis of PCa in the dog due to several factors including (a) the lack of early detection of sub‐clinical cases which are detected through prostate‐specific antigen (PSA) screening in men; (b) clinicians are unable to perform biopsies in asymptomatic dogs, and (c) misdiagnosis when advanced metatases in the lumbar spine and/or pelvis present as hindlimb pain and ataxia (LeRoy & Northrup, 2009; Leav & Ling, 1968).

As in men, canine BPH is an age‐related spontaneous disease process (Johnston et al., 2000). BPH is defined as an increase in the overall size of the prostate which is caused by hypertrophy (increase in size) and hyperplasia (increase in number) of epithelial cells (Lévy et al., 2014). Although the exact pathogenesis of BPH is not fully understood, several factors are clear: (a) the incidence increases with age; (b) it is an androgen‐dependent disease with higher concentrations of the androgen dihydrotestosterone (DHT) seen in hyperplastic compared with normal tissue; (c) presence of functioning testes is required (Wolf et al., 2012). The incidence and progression of BPH markedly increases with age. Half of dogs aged 4 years or more will have histological signs of BPH, increasing to over 90% by 8 years of age (Christensen, 2018). Importantly, DHT is the main mediator for the disease process, which causes glandular hyperplasia initially but then progresses to cystic hyperplasia. This cystic hyperplasia leads to what is known as a ‘honeycomb’ appearance histologically and can predispose to other prostatic diseases (Smith, 2008). Most dogs with BPH are asymptomatic but when clinical signs are seen these are most often haemospermia or urinary signs (incontinence, haematuria, stranguria), or gastrointestinal such as tenesmus and caudal (inferior) abdominal or generalised pain (Ravicini et al., 2018). When clinical signs are present, BPH is often referred to as ‘complicated hyperplasia’ (Foster, 2012). Of those dogs experiencing clinical signs, treatment focuses on reducing circulating DHT levels and, therefore, the size of the prostate. This is most frequently done by surgical or chemical castration and a significant reduction in prostatic size is usually seen within a few weeks. Chemical castration can be a preferred option in cases where the costs and risks associated with surgery are deemed too high, and in the UK is commonly achieved via monthly subcutaneous injection of delmadinone, a progestin anti‐androgen (Argyle et al., 2017) or using the depot gonadotrophin releasing hormone (GnRH) agonist, deslorelin.

PCa accounts for 5–7% of dogs that present with prostatic disease (Memon, 2007). The incidence of canine PCa is estimated to be less than 1% of canine malignancies, with reported rates between 0.45 and 0.93% (Bryan et al., 2007; Schrank & Romagnoli, 2020). Thus, the incidence of canine PCa is considerably lower than the 30% incidence reported in men, where PCa is the fourth leading cause of cancer‐related deaths (Schrank & Romagnoli, 2020; Siegel et al., 2020; Weaver, 1981). Of PCa seen in dogs, prostatic glandular and urothelial (transitional cell) carcinoma are the most common cancers arising from prostatic acini and urethra, respectively (Smith, 2008). In the recent years, canine PCa cell lines, including from brain metastases, have been successfully derived from dogs with spontaneous PCa (Elshafae et al., 2020; Packeiser et al., 2020; Simmons et al., 2014; Thudi et al., 2011), enabling a better understanding of the molecular characteristics of the disease in dogs.

**FIGURE 5:**
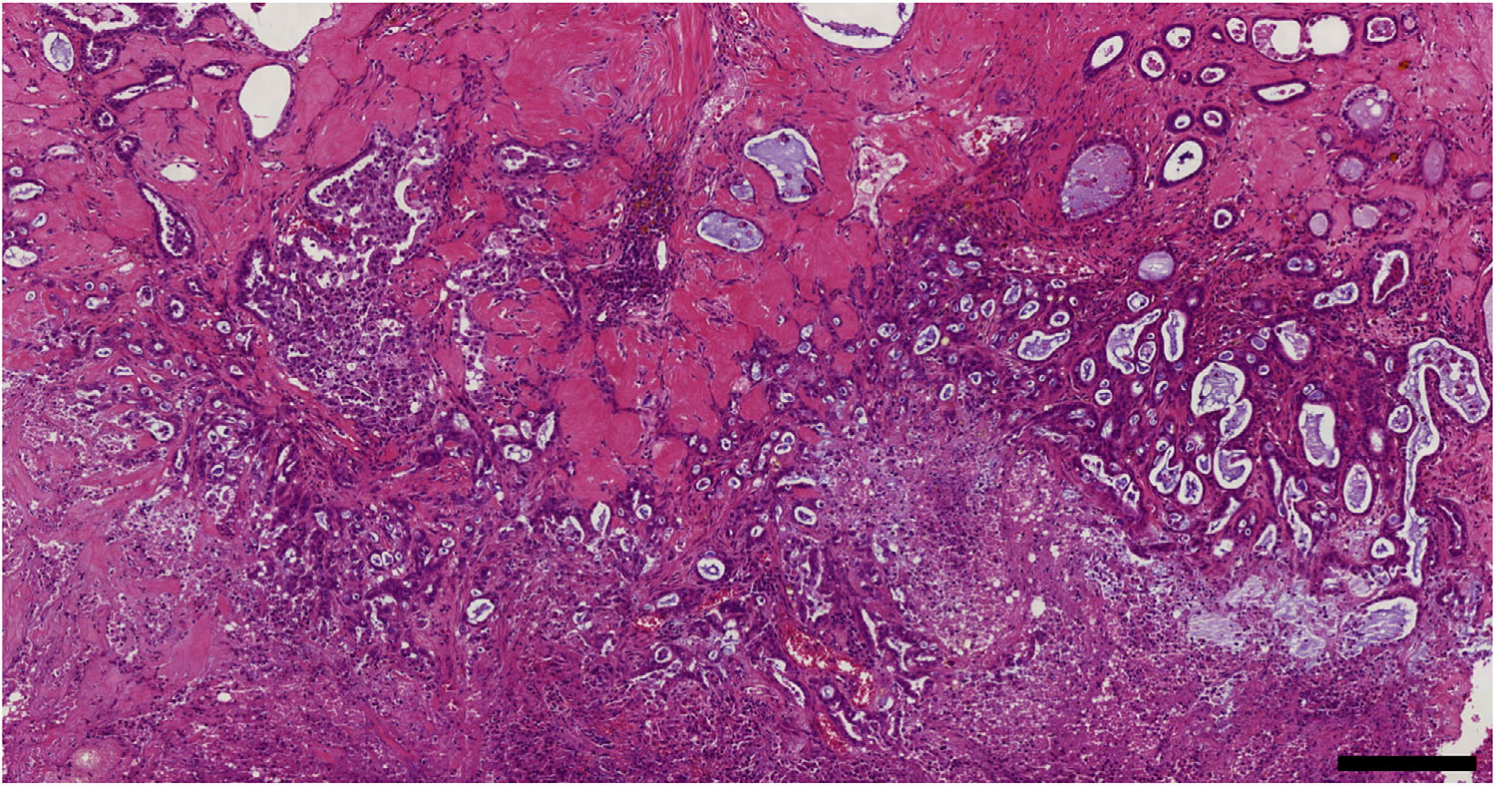
Canine glandular prostate carcinoma. Highly invasive growth and extensive necrosis are evident. HE stain. Size bar indicates 200 μm

**FIGURE 6:**
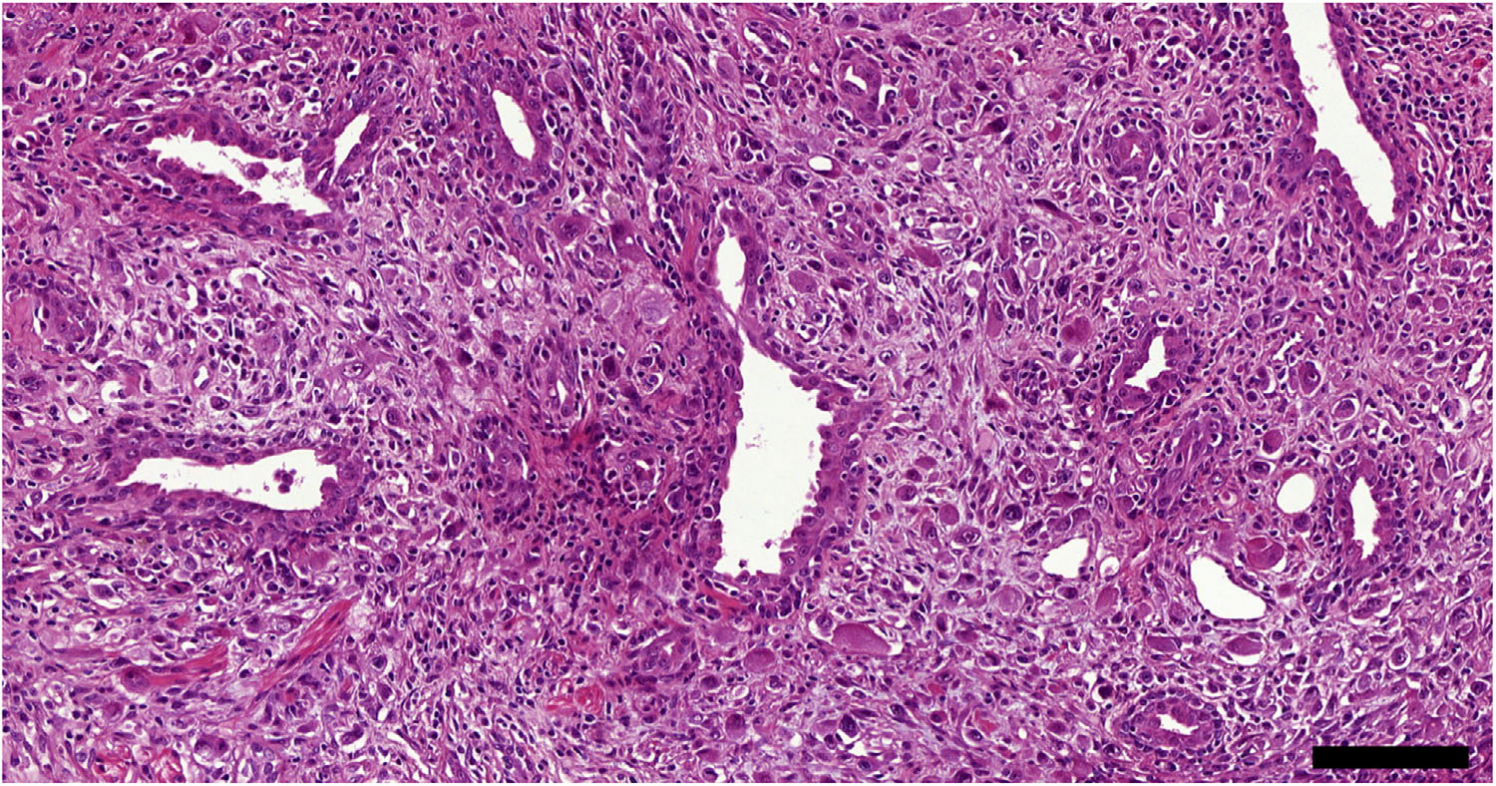
Canine poorly differentiated prostate carcinoma. Highly pleomorphic, frequently individualized neoplastic cells invade the stroma. HE stain. Size bar indicates 100 μm

Canine prostate carcinoma displays morphological heterogeneity with variable glandular differentiation making histopathological tumour classification difficult (Cornell et al., 2000; Lai et al., 2008) (Figures 5 and 6). PCa is commonly seen in older dogs with the mean age at diagnosis being 10 years old (Cornell et al., 2000). The clinical presentation of affected dogs is similar to that of other prostatic diseases including caudal abdominal pain, tenesmus and most commonly, dysuria (Palmieri et al., 2014). The metastasis rate of canine PCa is variable but can be as high as 40% at the time of diagnosis and 80% at the time of death (Cornell et al., 2000; Ravicini et al., 2018). Scosyrev et al. (2012) reported a metastasis rate of 3% in men under 75 years of age diagnosed with PCa. Cornell et al. (2000) reported, in the population of dogs that displayed gross metastasis, that the most common sites were lymph nodes (51%), lungs (50%) and bone (22%). Brain metastases, though rare in both men and dogs, are the most clinically serious with median overall survival after brain metastasis detection being less than 3 months (Bubendorf et al., 2000; Hatzoglou et al., 2014). The most frequently seen metastasis sites in human PCa are bone (84%), distal lymph nodes (10.6%) and liver (10.1%) (Cornell et al., 2000; Gandaglia et al., 2014; Keller et al., 2013). The prognosis of dogs that present with PCa is grave with reporting a mean survival time of only 17 days without treatment (Sorenmo et al., 2003), although this is likely to be influenced by late symptomatic presentation. This is vastly different from the prognosis seen in men, where the 5‐year incidence of death as a result of PCa in patients under 75 years of age has been reported to be as low as 3–4% (Scosyrev et al., 2012). This may, in part, be attributable to earlier detection and the availability of more advanced treatments in men. The use of PSA screening in dogs is controversial and is not used (Bell et al., 1995; Lai et al., 2008). However, the most abundant androgen‐dependant protein present in prostatic secretions of dogs is the canine prostatic specific esterase (CPSE) (Chapdelaine et al., 1984; Frenette et al., 1987). CPSE is not specific to canine PCa and is frequently present in other prostatic pathologies, therefore CPSE is unlikely to be a useful diagnostic marker (Alonge et al., 2018).

## 5 TREATMENT OF PCA IN MEN AND DOGS

**FIGURE 7:**
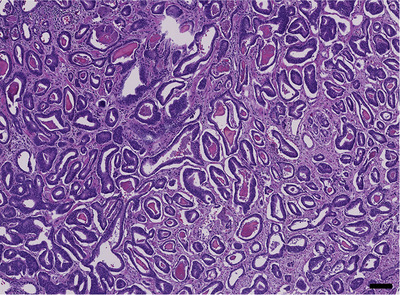
Human prostate carcinoma. Neoplastic glands are relatively uniform and well formed, compatible with Gleason pattern 3. The Gleason score, ranging from 1–5, is a grading system based on the histological characterisation of the glandular differentiation of prostate cancer, with higher scores associated poorer differentiation, more aggressive tumours and poor prognoses (Gleason & Mellinger, 1974; Humphrey, 2004). HE stain. Size bar indicates 100 μm

For men with PCa (Figure 7), treatment selection is based on the clinical and pathological grade of the tumour and the health of the patient. For patients with localised PCa or older patients, active surveillance may be a preferred option to avoid treatment and its associated adverse side‐effects (Klotz, 2010). Active surveillance involves regular tests like PSA screening, biopsies and magnetic resonance imaging (MRI) and aims to cure the cancer if treatment is given. In contrast, ‘watchful waiting’ is generally offered to men with either localised or advanced PCa who have other health problems (Coen et al., 2011). In general, localised PCa (stage 1) is treated by surgical removal of the prostate gland (prostatectomy), external radiotherapy or brachytherapy (D'Amico et al., 1998). Locally advanced and metastatic PCa is treated by systemic treatments such as hormonal therapy, immunotherapy and chemotherapy. As noted earlier, the most common treatment for patients with advanced disease is ADT. CRPC patients often receive chemotherapy, primarily docetaxel, a semi‐synthetic taxane that stabilises microtubules, thereby interfering with the mitotic spindle apparatus inducing cytotoxicity and apoptosis (Fauzee et al., 2011; Petrylak et al., 2004).

As the prognosis of canine PCa is poor and there have been limited advances in the management of the disease in dogs, information regarding effective treatment protocols is limited. The main aims of treatment in most cases are palliative, providing symptomatic relief and delaying the spread of the disease to maintain the quality of life for the dog. There are various approaches available, including surgery and medical treatments, which aim to help relieve clinical signs (Bennett et al., 2018; Ravicini et al., 2018). Bennett et al. (2018) showed an increase in survival time of dogs that underwent prostatectomy and adjuvant non‐steroidal anti‐inflammatory drugs (NSAIDs) and/or chemotherapy. However, this was influenced by a selected population with no metastases visible at the time of surgery and a high rate of post‐operative complications were seen (Bennett et al., 2018). NSAID treatment alone has been shown to reduce some of the clinical signs such as stranguria and faecal tenesmus (Sorenmo et al., 2004). Castration, either surgical or chemical, reduces androgen production and, hence, induces prostatic atrophy and involution but only in the non‐neoplastic regions of the prostate (Johnston et al., 2000). Treatment with NSAIDs and/or chemotherapy does give a longer mean survival time, however, prognosis remains guarded as this is only extended to 106 days (Ravicini et al., 2018).

## 6 EFFECTS OF CASTRATION ON CANINE PCa AND POTENTIAL MECHANISTIC SIMILARITIES TO CRPC IN MEN

Canine castration is common in developed countries and is done to prevent unwanted breeding and sexual behaviour as well as preventing androgen‐dependent diseases such as BPH (Fernando Leis‐Filho & Fonseca‐Alves, 2019). The effect of castration (neutering) on the incidence and progression of canine PCa has been investigated in many different studies. Many early studies concluded there was no evidence to suggest that castration affects the occurrence or progression of the disease (Bell et al., 1991; L'Eplattenier et al., 2006; Obradovich et al., 1987). In contrast, two studies reported an increased odds ratio of approximately 3.9 of prostatic neoplasia occurring in a neutered dog population compared to an entire population (Bryan et al., 2007; Sorenmo et al., 2003). Cornell et al. (2000) reported that castration had no effect on the occurrence of the disease but hypothesised that it plays an influential role in the progression of the disease from an androgen‐dependent to an androgen‐independent state. Similarly, other studies have reported that while castration does not initiate the neoplasia it can promote progression (Bryan et al., 2007; Cornell et al., 2000; Lai et al., 2008). Bell et al. (1991) reported an increase in the metastatic nature of PCa in castrated dogs with a larger proportion of castrated dogs having pulmonary metastases than entire dogs. This was further supported with the transgenic adenocarcinoma prostate (TRAMP) model that demonstrated that mice castrated at 12 weeks of age were more likely to develop lymph node metastases and a poorly differentiated cancer type (Gingrich et al., 1997). Teske et al. (2002) reported that while age of castration did not affect PCa initiation in the dog, it may influence disease progression. However, a more recent review (Schrank & Romagnoli, 2020) of the effects of castration on the development and progression of canine PCa concluded that further studies are required to address the combined effects of age at castration and the time interval from castration to diagnosis on both canine PCa risk and disease progression. Such knowledge may also improve the understanding of CRPC in men, where the age of diagnosis and duration of ADT in men may be a critical determinant of outcome.

## 7 CONCLUSIONS

The similarities of both prostate anatomy and disease in the dog make the canine species an important model in aiding our understanding of the progression of PCa in men. It is clear that more evidence is needed to elucidate the effects castration has on the acceleration of canine PCa. However, this further understanding of the effects of androgen depletion on PCa development and progression in the dog could provide insight into the deadly disease of CRPC in men.

## AUTHORS’ CONTRIBUTION

Toby Ryman‐Tubb: writing‐original draft; writing‐review & editing. Jennifer H. Lothion‐Roy: writing‐original draft; writing‐review & editing. Anna E. Harris: Writing‐original draft; Writing‐review & editing. Brian D. Robinson: Conceptualization; Writing‐original draft; writing‐review & editing. Jennie N. Jeyapalan: writing‐original draft; writing‐review & editing. Victoria H. James: writing‐original draft; writing‐review & editing. Gary England: writing‐original draft; writing‐review & editing. Catrin S. Rutland: writing‐original draft; writing‐review & editing. Jenny L. Persson: writing‐original draft; writing‐review & editing. Lukas Kenner: writing‐original draft; writing‐review & editing. Simone de Brot: conceptualization; formal analysis; supervision; writing‐original draft; writing‐review & editing.

## ETHICS STATEMENT

This study was reviewed and approved by the University of Nottingham School of Veterinary Medicine and Science ethics committee (approval numbers = 3246 200106 and 1669 160208).

## CONFLICT OF INTEREST

The authors declare no conflict of interests.

## DATA SHARING STATEMENT

Data sharing is not applicable to this article as no new data were created or analysed in this study.

## Data Availability

The authors have provided the required Data Availability Statement, and if applicable, included functional and accurate links to said data therein.
